# Improper Coordination of BamA and BamD Results in Bam Complex Jamming by a Lipoprotein Substrate

**DOI:** 10.1128/mBio.00660-19

**Published:** 2019-05-21

**Authors:** Muralidhar Tata, Anna Konovalova

**Affiliations:** a Department of Microbiology and Molecular Genetics, McGovern Medical School, The University of Texas Health Science Center at Houston (UTHealth), Houston, Texas, USA; University of Michigan-Ann Arbor

**Keywords:** Gram-negative envelope biogenesis, Rcs phosphorelay, surface-exposed lipoproteins

## Abstract

The β-barrel assembly machinery, the Bam complex, is central to the biogenesis of integral outer membrane proteins (OMPs) as well as OMP-dependent surface-exposed lipoproteins, such as regulator of capsule synthesis protein F (RcsF). Previous genetic analysis established the model that nonessential components BamE and BamB have overlapping, redundant functions to enhance the kinetics of the highly conserved BamA/BamD core. Here we report that BamE plays a specialized nonredundant role in the Bam complex required for surface exposure of RcsF. We show that the lack of *bamE*, but not *bamB*, completely abolishes assembly of RcsF/OMP complexes and establish that the inability to assemble RcsF/OMP complexes is a molecular reason underlying all synthetic lethal interactions of *ΔbamE*. Our genetic analysis and biochemical cross-linking suggest that RcsF accumulates on BamA when BamA cannot engage with BamD because of its limited availability or the incompatible conformation. The role of BamE is to promote proper coordination of RcsF-bound BamA with BamD to complete OMP assembly around RcsF. We show that in the absence of BamE, RcsF is stalled on BamA, thus blocking its function, and we identify the lipoprotein RcsF as a bona fide jamming substrate of the Bam complex.

## Abstract (precis)

The β-barrel assembly machinery, the Bam complex, consists of five components, BamA to -E, among which BamA and BamD are highly conserved and essential. The nonessential components are believed to play redundant roles simply by improving the rate of β-barrel folding. Here we show that BamE contributes a specific and nonoverlapping function to the Bam complex. BamE coordinates BamA and BamD to form a complex between the lipoprotein RcsF and its partner outer membrane β-barrel protein, allowing RcsF to reach the cell surface. In the absence of BamE, RcsF accumulates on BamA, thus blocking the activity of the Bam complex. As the Bam complex is a major antibiotic target in Gram-negative bacteria, the discovery that a lipoprotein can act as a jamming substrate may open the door for development of novel Bam complex inhibitors.

## INTRODUCTION

The heteropentameric β-barrel assembly machinery, the Bam complex, plays a central role in the outer membrane (OM) biogenesis in Gram-negative bacteria by promoting folding and insertion of integral β-barrel OM proteins (OMPs) (1). Although the overall function of the Bam complex is well established, the mechanistic contribution of its individual components (BamA to -E) is not well understood. BamA and BamD have emerged as the core components because of their essential nature and the high degree of conservation (2, 3). Genetic and biochemical evidence suggests that BamD recruits incoming OMP substrates (4, 5), while BamA is considered central for OMP folding/membrane integration due to its transmembrane nature (3, 6). The function of BamB, -E, and -C remains elusive. These components are not essential and are conserved in some, but not all, Gram-negative bacteria (7); therefore, they are unlikely to contribute a fundamental function to OMP assembly and are viewed as accessory components to enhance the kinetics of the BamAD core. Several lines of evidence support this model. Null mutations in *bamB* affect assembly of high-volume OMP substrates, such as porins, but do not affect the assembly of less abundant, and often more complex OMPs, including LptD and TolC (8–10). BamB is required for full efficiency of OMP assembly *in vitro* (11–13). Deletions of *bamE* and *bamC* do not affect the assembly of any single OMP; the most notable phenotype of *bamE* is its synthetic lethality at the physiological temperature (37°C) when combined with other *bam* mutations, including *bamB* (14–16). The *bamE bamB* double mutant can only grow either when the demand for the Bam complex efficiency is lowered under conditions of slow growth (minimal medium at 30°C) (15) or when the highly active σE stress response minimizes the periplasmic accumulation of toxic unfolded OMP substrates (17, 18). This observation led to the idea that BamE and BamB have overlapping, redundant functions supporting high efficiency of the OMP assembly under conditions of rapid growth (15).

In pursuit of the molecular mechanism of signal transduction by the Rcs stress response, we discovered that the Escherichia coli OM lipoprotein RcsF adopts a transmembrane topology by spanning the lumen of OMPs, most commonly OmpA (19). The N-terminal surface-exposed domain of RcsF is anchored in the outer leaflet of the OM by a lipid moiety to monitor disruptions in LPS packing (19, 20). Once stress is detected, the signal is transduced via the lumen of an OMP to the periplasmic C-terminal folded domain of RcsF to activate downstream signaling (20), releasing IgaA inhibition of the RcsCDB phosphorelay (21, 22). In response, RcsB acts as a homodimer or as a heterodimer with RcsA to regulate gene expression and promote envelope adaptation to stress (22).

Using a combination of *in vitro* and *in vivo* approaches, we previously demonstrated that the OMP barrel is folded around RcsF and that this reaction is catalyzed by the Bam complex *in vivo.* We thus uncovered a novel function of the Bam complex in the biogenesis of surface-exposed lipoproteins (19). We also showed that a *bamE* deletion completely abolishes assembly of RcsF/OMP complexes (20). Therefore, the RcsF/OMP complex is the first-described substrate of the Bam complex that requires BamE activity, suggesting that specialized activities of the Bam complex are needed for the assembly of this more challenging substrate. In the present study, we used the RcsF/OMP assembly as a tool to probe BamE function. Using genetic analysis and biochemical cross-linking, we demonstrate that RcsF is recruited to the Bam complex via BamA and that BamE plays a specialized, nonredundant role in coordinating lipoprotein/BamA and OMP/BamD core components to complete RcsF/OMP assembly. In the absence of BamE, RcsF accumulates on BamA, reducing the functional pool of Bam complexes in the cell. *ΔrcsF* is a potent suppressor of all *bamE* synthetic lethal interactions, restoring growth and OMP assembly in the otherwise lethal *bamE* double mutant backgrounds. Therefore, the lipoprotein RcsF represents a jamming substrate of the Bam complex.

## RESULTS

### RcsF inhibits the function of the Bam complex in the *ΔbamE* background.

**FIG 1:**
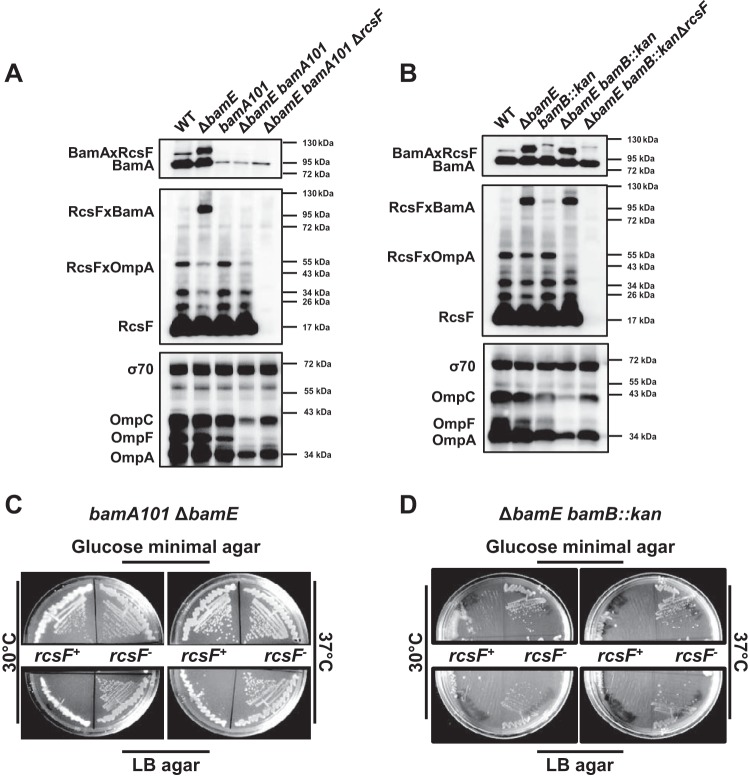
BamE plays a specific nonredundant role in the Bam complex required for the RcsF/OMP assembly. *ΔbamE* and not *bamA101* (A) or *bamB*::Kan (B) mutations result in the significant decrease of RcsF/OMP cross-linking. *ΔrcsF* improves OMP assembly in the corresponding double mutants (A and B, lower panels). Strains were grown in glucose minimal medium at 30°C, subjected to formaldehyde cross-linking, and analyzed by immunoblotting using anti-RcsF and anti-BamA antibodies. Immunoblot analysis of the total OMPs and σ70 (loading control) levels was performed on the total cell extracts (without cross-linking). Plate growth phenotype of *bamA101* Δ*bamE* (C) and *ΔbamE bamB*::Kan (D) double mutants and their *ΔrcsF* derivatives. Strains were streaked on indicated agar plates; plates were incubated at 30°C or 37°C. Growth was assayed after 48 h.

**FIG 2:**
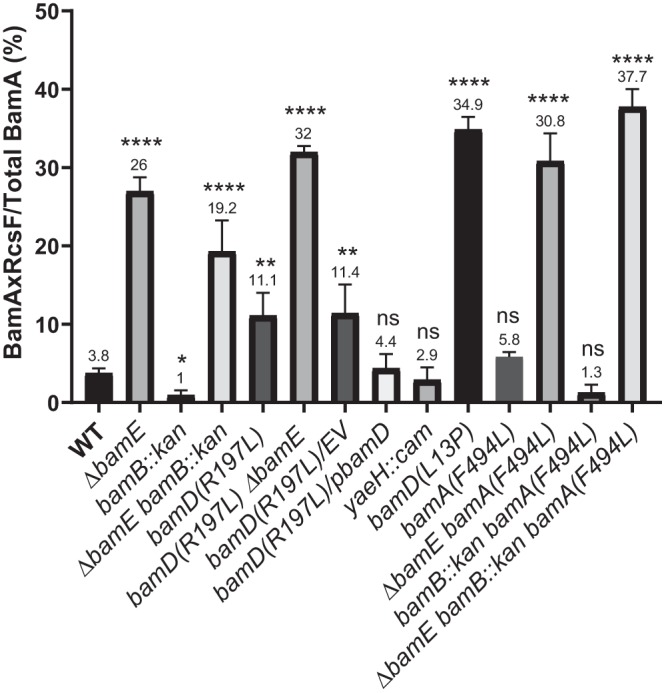
Quantitative analysis of the BamA fractions cross-linked to RcsF. Strains were grown and treated as described in the legend to Fig. 1. The intensity of BamA and BamAxRcsF bands was quantified using GelQuantNet software. Graphs represent mean fraction of BamAxRcsF as a percentage of total BamA ± standard error of the mean (SEM) based on at least three independent biological replicates. Significance analysis was performed using unpaired *t* test by comparing all strains with the WT. The asterisks represent *P* < 0.0001 (****), *P* < 0.001 (**), and *P* < 0.02 (*). For individual immunoblots, and their quantification, refer to Fig. S1 to S6 and Table S1.

We previously reported that BamE is required for assembly of RcsF/OMP complexes. In the *ΔbamE* strain, the reduction of RcsF/OMP cross-linking (estimated molecular weight of 50 kDa) is accompanied by the increase of RcsF/BamA cross-linking (estimated molecular weight of 110 kDa) (Fig. 1), suggesting that RcsF is arrested on BamA when the complex assembly is blocked. Here we began to elucidate the consequence of RcsF arrest on Bam complex function. We observed that around 30% of BamA is sequestered with RcsF in the *ΔbamE* strain as judged by the formaldehyde cross-linking (Fig. 1 and 2); however, this fraction is likely to be higher because cross-linking is not performed to saturation. Although this fraction is not sufficient to cause an obvious OMP assembly defect in the *ΔbamE* mutant, we reasoned that strains with lower levels or activity of BamA would be more sensitive to RcsF-dependent BamA sequestration, which may explain the well-documented conditional synthetic lethality of *ΔbamE* when combined with *bamA101* or *bamB* mutations (14, 15). The *bamA101* mutation harbors a Tn*5* insertion in the promoter region of *bamA*, decreasing BamA levels approximately 10-fold (23). Unlike the *bamA101* mutation, a *bamB* mutation does not affect BamA levels but confers a general OMP assembly defect due to partially compromised Bam complex activity (10). Accordingly, the *bamB* strain also cannot tolerate a further reduction in BamA level or activity, and *bamB* is essential in the *bamA101* background at temperatures above 24°C (Table 1).

**TABLE 1:** Plate growth phenotype of the OMP assembly-defective double mutants^*a*^

Mutation	Growth of strain in medium:					
	Glucose minimal medium			LB		
	Parent	*ΔrcsF*	*ΔrcsB*	Parent	*ΔrcsF*	*ΔrcsB*
Δ*bamE bamB*::Kan	TS@37	+	TS@37	TS@30	+	TS@30
Δ*bamE bamA101*	+	+	TS@30	TS@30	+	TS@30
*bamA101 bamB8*^*b*^	TS@30	TS@30	ND	−	−	ND
*degP*::Cm *bamB*::Kan	+	+	ND	TS@37	TS@37	ND
*bamE*::Cm *bamD*(*R197L*)	+	+	ND	TS@37	+	ND

a The strains were assayed for the ability to grow and form isolated colonies on top of the solid agar plates at 24°C, 30°C, and 37°C. The temperature-sensitive (TS) phenotype is indicated by the lowest temperature (°C) at which growth was no longer observed. The + and − signs indicate growth or lack of growth, respectively, at all temperatures tested; ND, not determined.

b *bamB8* is a markerless null allele of *bamB* (46). It was used to assay *bamA101* interaction due to the incompatible antibiotic resistance marker of *bamB*::Kan.

10.1128/mBio.00660-19.1 FIG S1 Independent biological replicates of RcsFxBamA cross-linking experiments presented in Fig. 1A. Download FIG S1, PDF file, 0.7 MB. Copyright © 2019 Tata and Konovalova. 2019 Tata and Konovalova This content is distributed under the terms of the Creative Commons Attribution 4.0 International license.

10.1128/mBio.00660-19.8 TABLE S1 Quantitative analysis of the BamA fractions cross-linked to RcsF based on individual immunoblots from independent biological replicates. Download Table S1, PDF file, 0.3 MB. Copyright © 2019 Tata and Konovalova. 2019 Tata and Konovalova This content is distributed under the terms of the Creative Commons Attribution 4.0 International license.

To test our hypothesis, we first constructed *ΔbamE bamA101* and *ΔbamE bamB* double mutants with or without *ΔrcsF* under permissive condition (glucose minimal medium at 30°C) and tested these mutants for RcsF/OMP assembly. Unlike *ΔbamE*, neither *bamA101* nor *bamB* affected RcsF/OMP assembly (Fig. 1A and B), demonstrating a specific role of BamE in this process. As expected, the introduction of Δ*bamE* into either the *bamA101* or *bamB* background resulted in a significant reduction of RcsF/OMP cross-linking (Fig. 1A and B). The Δ*bamE bamB* strain largely phenocopied Δ*bamE,* and RcsF accumulated on BamA. We were not able to detect the RcsF/BamA cross-link in either of the *bamA101* strains, likely because BamA levels are significantly reduced (Fig. 1A and B).

According to our hypothesis, RcsF sequestration could reduce the functional pool of BamA to lethal levels in the double mutants. In strong support of this hypothesis, we determined that the temperature-sensitive phenotype of *ΔbamE bamA101* and Δ*bamE bamB* double mutants is *rcsF* dependent (Table 1; Fig. 1C and D). *ΔrcsF* restored growth of these double mutants on LB at 30°C and 37°C, although the Δ*bamE bamB ΔrcsF* strain grew slower than the wild type (WT) on LB agar at 37°C. Consistent with the growth phenotype, we also observed that the introduction of *ΔrcsF* improved OMP assembly in *ΔbamE bamA101* (Fig. 1A, compare the last two lanes) and *ΔbamE bamB* (Fig. 1B, compare the last two lanes) double mutants, although it was not restored to the WT levels.

Growth under low temperatures and on minimal glucose medium leads to increased Rcs activity evident by the mucoid phenotype. This phenomenon is poorly understood but is explained at least in part by the increased levels of RcsA (22). Because Rcs activity can be toxic (21, 24) and *ΔrcsF* inactivates the pathway, we tested whether inactivation of Rcs by *ΔrcsB* would also suppress *ΔbamE bamA101* and *ΔbamE bamB* double mutants. We found that unlike *ΔrcsF*, *ΔrcsB* could not restore growth on LB; moreover, *ΔrcsB* resulted in more severe growth defects in the double mutants (Table 1). This result demonstrates that the suppression by *ΔrcsF* is independent of RcsF signaling function. Finally, the *ΔrcsF* mutation is not a general suppressor of OMP assembly mutants, because it is unable to suppress the *bamA101 bamB* and *bamB degP* (10) synthetic lethal pairs (Table 1).

Based on the above results, we concluded that the inability to assemble RcsF causes the conditional essentiality of *bamE*. In the absence of *bamE*, RcsF remains on BamA, sequestering BamA from functioning in the OMP assembly.

### Proper engagement of BamA and BamD is critical for RcsF/OMP assembly.

The *ΔbamE* mutant also displays a synthetic lethal interaction with *bamD*(*R197L*) (25). *bamD*(*R197L*) is a gain-of-function mutation which enables BamD to function independently of the direct interaction with BamA. It was isolated as a suppressor of the lethal *bamA*(*E373K*) mutation that targets a salt bridge critical for BamA-BamD coordination (26, 27). However, the *bamE bamD*(*R197L*) synthetic lethal pair is distinct from those described above, because *bamD*(*R197L*) does not confer any detectable phenotype in an otherwise WT background (25, 27). This intriguing synthetic lethality led to a proposed conformational cycling model, in which both *bamE* and *bamD*(*R197L*) bias BamA toward a distinct conformation, preventing it from undergoing the normal dynamic cycle needed for efficient OMP assembly (25).

**FIG 3:**
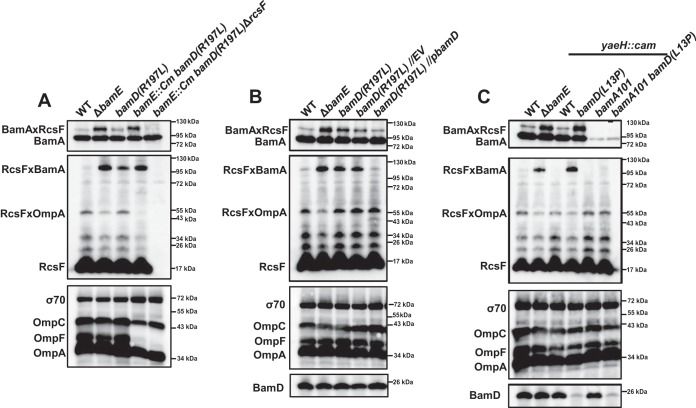
Improper coordination of BamA and BamD results in RcsF accumulation on BamA. (A) *bamD*(*R197L*) results in increased RcsF/BamA cross-linking. (B) The *bamD*(*R197L*) mutation is recessive to the WT *bamD* allele. (C) Decreased levels of BamD relative to BamA abolish RcsF/OMP assembly, leading to RcsF accumulation on BamA. Strains were grown in glucose minimal medium at 30°C, subjected to formaldehyde cross-linking, and analyzed by immunoblotting using anti-RcsF and anti-BamA antibodies. Immunoblot analysis of the total OMPs and σ70 (loading control) levels was performed on the total cell extracts (without cross-linking).

Deletion of *ΔrcsF* restored growth of the *bamE*::Cm *bamD*(*R197L*) double mutant on LB at 37°C (Table 1), demonstrating that *ΔrcsF* acts as a *bamE* suppressor and suppresses all *bamE* synthetic lethal combinations regardless of the underlying defect. Surprisingly, however, the cross-linking experiments revealed that the *bamD*(*R197L*) mutation also led to increased RcsF accumulation on BamA, although the phenotype was milder than that observed for the *bamE* mutant (Fig. 2 and 3A).

We next sought to investigate the underlying reason for the phenotype of the *bamD*(*R197L*) strain. We envisioned two possible scenarios by which the *R197L* mutation could affect the RcsF/OMP assembly. In the first scenario, R197L would bias BamD toward a conformation unable to engage with RcsF-bound BamA. In the second scenario, BamD(R197L) would engage with RcsF-bound BamA in an unproductive manner, arresting the RcsF/OMP assembly process. We used genetic analysis to differentiate between these scenarios. *bamD*(*R197L*) is expected to be recessive to the WT *bamD* allele in the former case, while in the latter case it would confer a dominant negative phenotype. To test for dominance, we introduced the WT copy of *bamD* on a low-copy-number pZS21 plasmid into the *bamD*(*R197L*) strain and performed biochemical cross-linking experiments (Fig. 3B). RcsF/BamA cross-linking was reduced back to the WT levels in the *bamD* merodiploid strain compared with the elevated levels in the empty vector (EV) control (Fig. 2 and 3B). This recessive nature of the *bamD*(*R197L*) mutation is indicative of its loss-of-function nature regarding RcsF/OMP assembly, which is in stark contrast to its gain-of-function nature for general OMP assembly (25, 26).

We next reasoned that if the inability of BamD(R197L) to engage with RcsF-bound BamA resulted in RcsF stalling on BamA, then the same phenotype would be observed when BamD was absent. Like *bamA*, *bamD* is essential (2), so we used *bamD*(*L13P*), a mutation in the signal sequence that causes inefficient export of BamD across the Sec translocon, resulting in an approximately 10-fold reduction in BamD levels (Fig. 3C) (8). Importantly, the mature BamD protein still has the WT sequence in this case. Like *bamA101*, *bamD*(*L13P*) affects the efficiency of the OMP assembly by an overall reduction in the number of functional Bam complexes (8). However, *bamD*(*L13P*) results in a phenotype distinct from *bamA101*, as no RcsF/OMP complexes were formed and RcsF accumulated on BamA (Fig. 2 and 3C). Importantly, the *bamA101 bamD*(*L13P*) double mutant, in which the ratio of BamA and BamD is restored, showed restoration of RcsF/OMP assembly (Fig. 2 and 3C). Based on these results, we concluded that RcsF accumulates on BamA when BamA cannot engage with BamD either because of its limited availability [e.g., *bamD*(*L13P*)] or because BamD is in an incompatible conformation [e.g., *bamD*(*R197L*)]. Because both of the *bamD* mutant strains phenocopied *ΔbamE*, we concluded that the underlying defect of *ΔbamE* is also related to BamA/BamD engagement.

**FIG 4:**
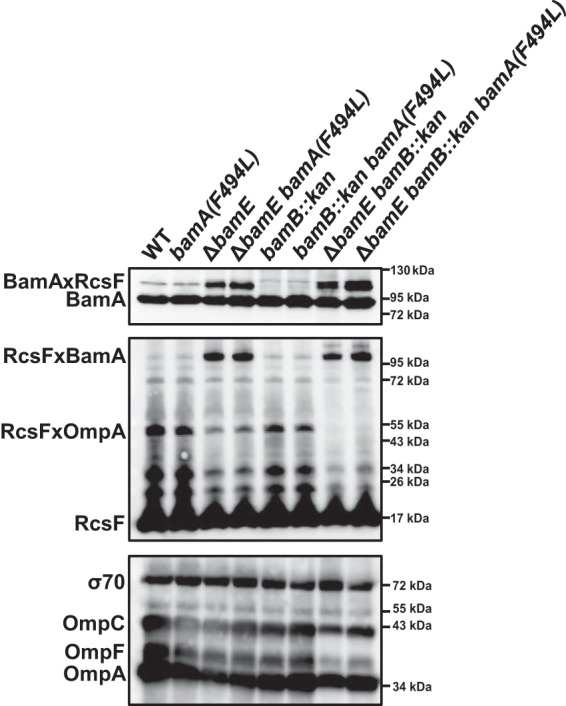
The *bamA*(*F494L*) mutation does not restore the RcsF/OMP assembly in the *ΔbamE bamB* strain. Strains were grown in glucose minimal medium at 30°C, subjected to formaldehyde cross-linking, and analyzed by immunoblotting using anti-RcsF and anti-BamA antibodies. Immunoblot analysis of the total OMPs and σ70 (loading control) levels was performed on the total cell extracts (without cross-linking).

Several *bamA* suppressors that enable growth of the *ΔbamE bamB* strain under nonpermissive conditions have been previously isolated (15). One such mutation, *bamA*(*F494L*), is of particular interest. It is a gain-of-function mutation that also allows the cell to survive despite very low levels of BamD (28). This mutation also was independently isolated as a suppressor restoring the assembly of a defective OMP substrate, LptD(Y721D), which accumulates on BamD as a result of defective BamA/BamD coordination (4). We tested the effect of the *bamA*(*F494L*) mutation on RcsF cross-linking in single and double mutants (Fig. 4). We observed that *bamA*(*F494L*) did not change the RcsF cross-linking pattern in either of the strains, demonstrating that *bamA*(*F494L*) improves the OMP assembly defect without restoring RcsF/OMP assembly (Fig. 2 and 4).

## DISCUSSION

The RcsF/OMP complex is a novel type of Bam-dependent protein complex consisting of a lipoprotein and an OMP partner. In this complex, an OMP barrel is folded around the RcsF unstructured region, allowing it to adopt a transmembrane topology with its lipidated N-terminal domain exposed on the cell surface (19). Assembly of this complex requires some of the distinct activities of the Bam machinery, specifically, the ability to (i) simultaneously recognize both lipoprotein and OMP substrates, (ii) translocate the RcsF lipid moiety to the OM outer leaflet, and (iii) coordinate lipoprotein surface exposure with OMP assembly. Previous studies established that OMP substrates are recruited to the Bam complex via BamD (4, 5). Here we show that RcsF is recruited via BamA and independently of BamD. The progression of RcsF/OMP assembly requires BamE, which plays a specific role in coordinating RcsF-bound BamA with BamD. In the absence of BamE, RcsF remains stalled on BamA, preventing BamA from functioning in the OMP assembly, and thereby acting as a jamming substrate of the Bam complex.

BamE is not essential, and its function in the Bam complex is poorly studied, primarily due to the lack of a significant phenotype of *ΔbamE* in OMP assembly (14, 16). The most notable phenotype is the well-documented synthetic lethal interactions when *ΔbamE* is combined with other *bam* mutations, including *bamB* (14, 15). This observation led to the idea that BamE and BamB have overlapping, redundant functions to enhance the kinetics of the BamAD core components (15). Here we show that, unlike what was previously thought, BamE plays a specific nonredundant role in the Bam complex. BamE, but not BamB, is critical for the RcsF/OMP assembly. Moreover, RcsF-dependent sequestration of BamA is the molecular reason underlying synthetic lethal interactions of *ΔbamE*, including that with *bamB*. *ΔrcsF* acts as a suppressor of growth and OMP assembly in the *ΔbamE bamB* and *ΔbamE bamA101* double mutants. In the accompanying paper (29), Hart et al. use a quantitative proteomic analysis to demonstrate that the *bamE bamB* double mutant displays a broad *rcsF*-dependent OMP assembly defect. Removal of *rcsF* results in the global restoration of OMP levels nearly to the same extent as the previously characterized *bamA*(*F494L*) suppressor (4, 15, 28). This proteomic analysis is consistent with general inhibition of the Bam complex function by stalled RcsF.

Based on the results presented above, we proposed that BamE plays a specific role in RcsF/OMP assembly by promoting proper coordination of RcsF-bound BamA with BamD, likely bound to the OMP substrate. BamA/BamD coordination is essential for OMP assembly and cell growth. In WT cells, it involves conformational changes in BamA and BamD regulated through a direct interaction between the two proteins at the BamA Potra 5 domain interface (26, 27). *bamD*(*R197L*) is a gain-of-function mutation for OMP assembly, in which BamD is biased toward an altered conformation, bypassing the requirement for BamA-induced activation (26, 27). *bamD*(*R197L*) is compatible with both WT and otherwise-lethal *E373K* alleles of *bamA* (26, 27). In contrast, we showed that *bamD*(*R197L*) is a recessive, partial-loss-of-function mutation with regard to RcsF assembly, resulting in an increase in RcsF/BamA cross-linking, thus phenocopying the *ΔbamE* mutation. Importantly, the same phenotype is also observed under the conditions of BamD limitation in the *bamD*(*L13P*) strain, and therefore, we concluded that RcsF accumulates on BamA when BamA is not engaged with BamD.

BamE is clearly dispensable for BamA/BamD coordination during normal OMP assembly because it is not essential and does not confer an OMP assembly defect (14, 16). So why is BamE important for BamA/BamD coordination during RcsF/OMP assembly? We propose that RcsF binding to BamA alters BamA conformation in such a way that it is unable to directly engage with BamD and therefore requires BamE activity for this process. In the absence of BamE, BamA/BamD cannot communicate to complete OMP assembly around RcsF, and RcsF remains stalled on BamA. The RcsF-induced conformational change of BamA also causes incompatibility with BamD(R197L) regardless of the presence of BamE, which is reminiscent of the reciprocal genetic incompatibility of BamD(R197L) with BamA variants with an altered electrostatic network at the BamA-Potra 5/BamD interface (26). Based on the structure of the Bam complex, BamE interacts with both BamA and BamD (30, 31). More studies are needed to understand whether BamE coordinates BamA/BamD during RcsF/OMP assembly by regulating the conformation of BamA, BamD, or both.

Another two-partner lipoprotein/OMP complex in E. coli is LptE/LptD, which is an essential complex for LPS transport across the OM (32). BamE activity is not required for LptE/LptD assembly (14, 16). The fundamental difference between RcsF/OMP and LptE/LptD is the lipoprotein topology in the complex (19, 33–35). We think it is likely that a specific requirement for BamE in RcsF/OMP assembly is directly related to the ability of the Bam complex to translocate RcsF lipid moieties to the outer leaflet of the OM, leading to the surface exposure of the RcsF N-terminal domain.

Our studies of RcsF/OMP assembly led to the discovery of a novel function of the Bam complex in the biogenesis of surface-exposed lipoproteins. Deciphering the RcsF/OMP assembly pathway not only would be highly informative for a further mechanistic understanding of this versatile macromolecular machinery but might also open the doors for novel therapeutic development strategies. The Bam complex has emerged as a major antibiotic target due to its central and essential role in the biogenesis of the OM, the primary factor of intrinsic antibiotic resistance in Gram-negative bacteria (36–38). Previous studies have explored the possibility of using OMP-derived-peptides that mimic native OMP substrates to inhibit the Bam complex via BamD (5). However, both assembly-defective OMP substrates and OMP-derived peptides strongly activate the σE envelope stress response, which in turn induces their rapid degradation, thereby promoting cell survival (18, 39, 40). Our results showing that the Bam complex activity can be blocked by a lipoprotein rather than an OMP substrate may provide alternative or additional routes for Bam complex inhibition.

## MATERIALS AND METHODS

### Bacterial strains and growth conditions.

All the bacterial strains used in this study are derived from MC4100 (2) and are listed in Table S2 in the supplemental material. All the strains were constructed by generalized P1 transduction (41). The deletion alleles originated from the Keio collection (42), and the Kan cassettes were excised using the Flp recombinase (43). Strains were grown at either 37°C or 30°C as indicated. Lysogeny broth (LB)-Lennox or minimal glucose medium (26.1 mM Na_2_HPO_4,_ 22 mM KH_2_PO_4_, 8.5 mM NaCl, 18.6 mM NH_4_Cl, 0.2% glucose, 1 mM MgSO_4_, 100 μg/ml thiamine) was used as a growth medium. A final concentration of 100 μM β-NAD hydrate (Millipore Sigma) was added for the growth of *nadA*::Tn*10* and *nadB*::Tn*10* strains. When required, the following concentrations of antibiotics were used: 125 μg/ml ampicillin, 20 μg/ml tetracycline, 25 μg/ml kanamycin, and 20 μg/ml chloramphenicol.

### *In vivo* formaldehyde cross-linking and immunoblot analyses.

Cross-linking experiments were performed in at least three biological replicates. Strains were grown in a glucose minimal medium at 30°C to an OD_600_ of 0.7 to 1.2, washed twice in phosphate-buffered saline (PBS) (10 mM Na_2_HPO_4_, 1.8 mM KH_2_PO_4_, 2.7 mM KCl, 137 mM NaCl, pH 7.6) and normalized to an optical density (OD_600_) of 10 in PBS. Cell suspensions were split into two 200-μl samples; one was subjected to cross-linking, while the second sample was used to determine total levels of OMPs and BamD (see below).

Cross-linking was carried out in 200 μl of cell suspension by addition of formaldehyde to a final concentration of 0.7% for 12 min at room temperature. The reaction was stopped by addition of Tris-Cl (pH 6.8) to a final concentration of 100 mM. The cells of cross-linked and non-cross-linked samples were harvested by centrifugation and resuspended in 100 μl of BBB buffer (1× BugBuster reagent [Millipore Sigma], 50 mM Tris-Cl, pH 6.8, and 1 μl Benzonase [Millipore Sigma]). After incubation on the bench for 2 to 3 min, 100 μl of 2× SDS loading buffer was added and samples were heated at 65°C for 15 min.

For immunoblotting, 10 μl of samples, normalized by OD_600_ prior to cross-linking, was separated on SDS-PAGE. The proteins were blotted onto a polyvinylidene difluoride (PVDF) membrane and blocked with 2% nonfat dried milk in wash buffer (1.21 g/liter Tris base, 9 g/liter NaCl, 0.05% Tween 20). The membranes were probed with previously validated polyclonal rabbit antibodies raised against RcsF (1:10,000) (20); BamA (1:40,000) (3); OmpA, OmpC, and OmpF (1:20,000) (44, 45); BamD (1:5,000) (16); and σ70 (1:20,000). σ70 protein was served as a loading control. Donkey anti-rabbit IgG linked to HRP (1:10,000) (GE Healthcare) was used as a secondary antibody. Immunoblots validating band identities using deletions of corresponding nonessential genes are shown in Fig. S7.

10.1128/mBio.00660-19.2 FIG S2 Independent biological replicates of RcsFxBamA cross-linking experiments presented in Fig. 1B. Download FIG S2, PDF file, 0.7 MB. Copyright © 2019 Tata and Konovalova. 2019 Tata and Konovalova This content is distributed under the terms of the Creative Commons Attribution 4.0 International license.

10.1128/mBio.00660-19.3 FIG S3 Independent biological replicates of RcsFxBamA cross-linking experiments presented in Fig. 3A. Download FIG S3, PDF file, 0.7 MB. Copyright © 2019 Tata and Konovalova. 2019 Tata and Konovalova This content is distributed under the terms of the Creative Commons Attribution 4.0 International license.

10.1128/mBio.00660-19.4 FIG S4 Independent biological replicates of RcsFxBamA cross-linking experiments presented in Fig. 3B. Download FIG S4, PDF file, 0.7 MB. Copyright © 2019 Tata and Konovalova. 2019 Tata and Konovalova This content is distributed under the terms of the Creative Commons Attribution 4.0 International license.

10.1128/mBio.00660-19.5 FIG S5 Independent biological replicates of RcsFxBamA cross-linking experiments presented in Fig. 3C. Download FIG S5, PDF file, 0.7 MB. Copyright © 2019 Tata and Konovalova. 2019 Tata and Konovalova This content is distributed under the terms of the Creative Commons Attribution 4.0 International license.

10.1128/mBio.00660-19.6 FIG S6 Independent biological replicates of RcsFxBamA cross-linking experiments presented in Fig. 4. Download FIG S6, PDF file, 0.8 MB. Copyright © 2019 Tata and Konovalova. 2019 Tata and Konovalova This content is distributed under the terms of the Creative Commons Attribution 4.0 International license.

10.1128/mBio.00660-19.7 FIG S7 Validation of immunoblot band identities through a mutant analysis. Download FIG S7, PDF file, 0.2 MB. Copyright © 2019 Tata and Konovalova. 2019 Tata and Konovalova This content is distributed under the terms of the Creative Commons Attribution 4.0 International license.

The blots were developed with Luminata Crescendo Western HRP substrate (Millipore) and visualized using an ImageQuant LAS 4000 Mini (GE Healthcare). The intensity of BamA and BamAxRcsF bands was quantified using GelQuantNet software. Graphs were built using GraphPad Prism software. Significance analysis was performed using an unpaired *t* test by comparing all strains with the WT (using GraphPad Prism).

10.1128/mBio.00660-19.9 TABLE S2 Bacterial strains used in this study. Download Table S2, PDF file, 0.4 MB. Copyright © 2019 Tata and Konovalova. 2019 Tata and Konovalova This content is distributed under the terms of the Creative Commons Attribution 4.0 International license.
