# Supplementary material for: Improper Coordination of BamA and BamD Results in Bam Complex Jamming by a Lipoprotein Substrate
Source: mBio. 2019 May 21;10(3):e00660-19. doi: 10.1128/mBio.00660-19 (PMC6529637; doi:10.1128/mBio.00660-19)
Supplement: TABLE S1 [file mBio.00660-19-st001.pdf]

Table S1. Quantitative analysis of the BamA fractions cross-linked to RcsF based on individual immunoblots from independent biological replicates.
Strains were grown and treated as described in Fig. 1. Immunoblots from independent experiments were quantified using GelQuantNet software. Values represent a fraction of BamAxRcsF as a percentage of total BamA.

	**BamAxRcsF/Total BamA (%) Replicates**		
	**1**	**2**	**3**
	**Fig. S1-A**	**Fig.S1-B**	**Fig.S1-C**
WT	11.393	1.599	6.374
Δ*bamE*	36.181	17.139	34.819
	**Fig.S2-A**	**Fig.S2-B**	**Fig.S2-C**
WT	0.759	3.312	1.089
Δ*bamE*	21.501	20.340	13.605
bamB::kan	0.459	0.205	0.091
Δ*bamE bamB::kan*	15.080	14.511	10.804
	**Fig.S3-A**	**Fig.S3-B**	**Fig.S3-C**
WT	4.956	2.960	2.632
Δ*bamE*	29.775	33.444	34.546
bamD(R197L)	7.536	8.112	6.553
bamD(R197L) bamE::Cm	30.711	31.751	33.422
	**Fig.S4-A**	**Fig.S4-B**	**Fig.S4-C**
WT	3.492	4.038	1.220
Δ*bamE*	28.323	32.253	24.744
bamD(R197L)	22.374	17.467	4.524
bamD(R197L)//EV	14.616	15.525	4.048
bamD(R197L)//pbamD	4.864	7.248	1.012
	**Fig.S5-A**	**Fig.S5-B**	**S5-C**
WT	6.067	0.782	2.479
Δ*bamE*	27.444	14.546	21.892
yaeH::cam	5.900	0.425	2.362
pbamD(L13P)	36.087	31.664	36.797
	**Fig.S6-A**	**Fig.S6-B**	**Fig.S6-C**
WT	5.503	3.474	5.515
bamA(F494L)	6.055	4.664	6.784
Δ*bamE*	29.450	26.627	38.940
Δ*bamE bamA(F494L)*	31.143	24.545	36.783
bamB::kan	0.544	0.581	3.856
bamB::kan bamA(F494L)	0.432	0.683	3.271
Δ*bamE bamB::kan*	23.999	14.405	36.946
Δ*bamE bamB::kan bamA(F494L)*	40.325	33.206	39.680
