# Supplementary material for: Improper Coordination of BamA and BamD Results in Bam Complex Jamming by a Lipoprotein Substrate
Source: mBio. 2019 May 21;10(3):e00660-19. doi: 10.1128/mBio.00660-19 (PMC6529637; doi:10.1128/mBio.00660-19)
Supplement: FIG S4 [file mBio.00660-19-sf004.pdf]

Supplementary Figure-4

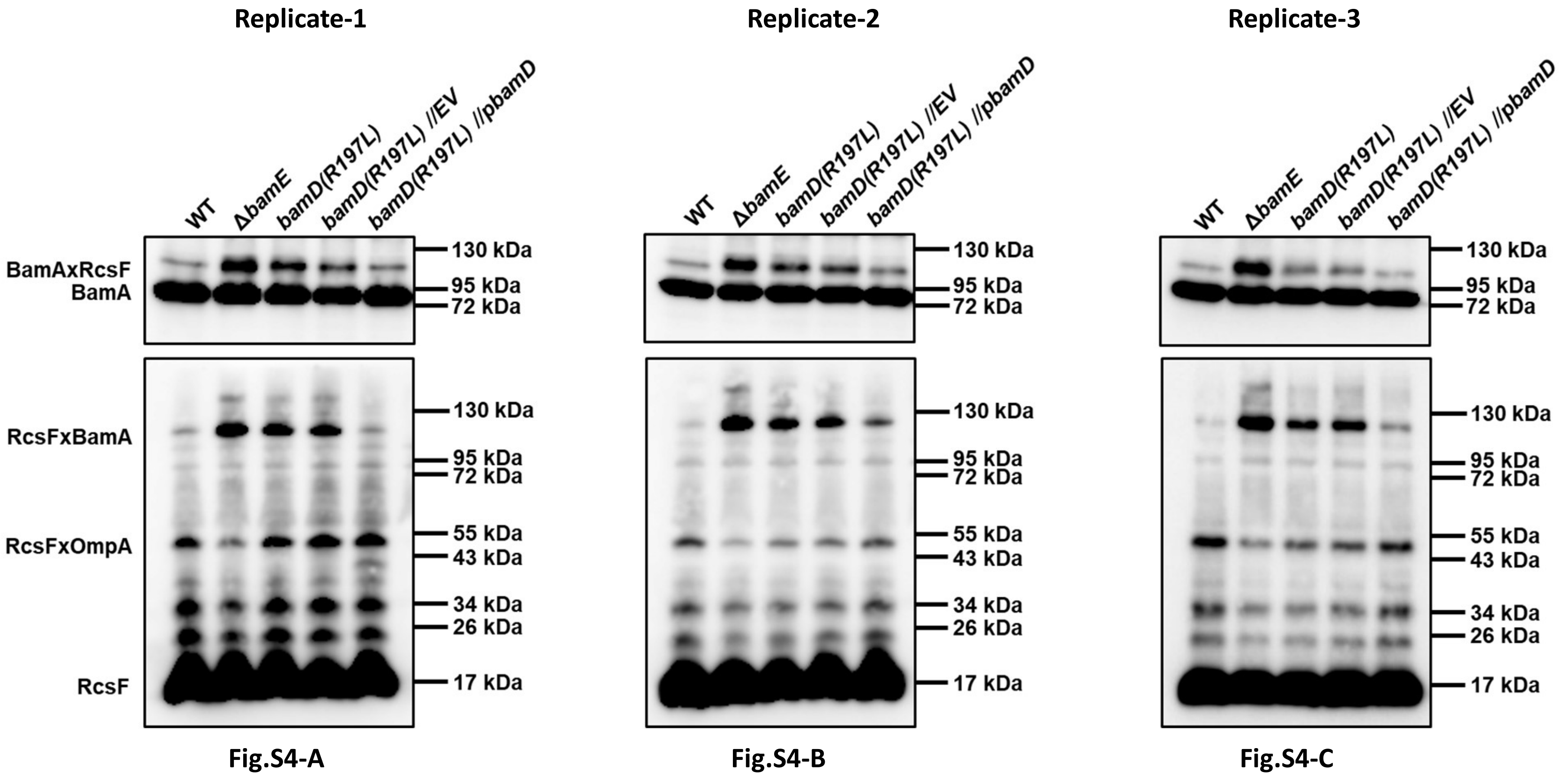

Independent biological replicates of RcsFxBamA cross-linking experiments presented in Fig. 3B.
Indicated strains were grown in glucose minimal media at 30 °C, subjected to a formaldehyde crosslinking and analyzed by immunobloting using anti-RcsF and anti-BamA antibodies. See Supplementary table 1 for quantifications of RcsFxBamA crosslinking bands.
