# Supplementary material for: Improper Coordination of BamA and BamD Results in Bam Complex Jamming by a Lipoprotein Substrate
Source: mBio. 2019 May 21;10(3):e00660-19. doi: 10.1128/mBio.00660-19 (PMC6529637; doi:10.1128/mBio.00660-19)
Supplement: FIG S7 [file mBio.00660-19-sf007.pdf]

Supplementary Figure-7

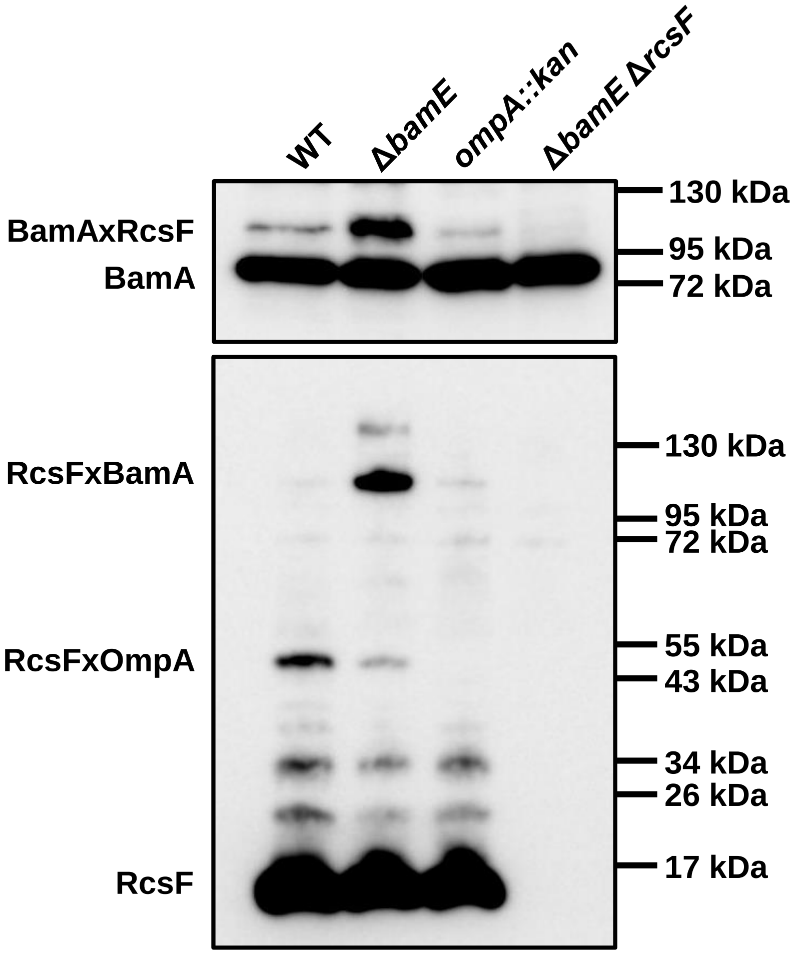

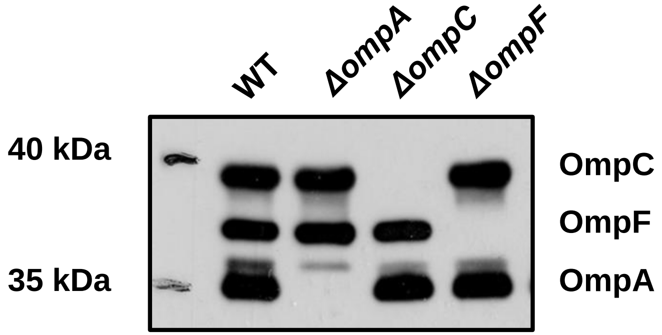

Validation of immunoblot band identities through a mutant analysis.

Indicated strains were grown in glucose minimal media at 30 ºC and subjected to a formaldehyde crosslinking and analyzed by immunobloting using anti-RcsF and anti-BamA antibodies. Immunoblot analysis of the total was performed on the total cell extracts (without crosslinking).
