# Supplementary material for: Improper Coordination of BamA and BamD Results in Bam Complex Jamming by a Lipoprotein Substrate
Source: mBio. 2019 May 21;10(3):e00660-19. doi: 10.1128/mBio.00660-19 (PMC6529637; doi:10.1128/mBio.00660-19)
Supplement: TABLE S2 [file mBio.00660-19-st002.pdf]

Supplementary Table-2. Strains used in this study.

Strain	Genotype	Annotation in Figures	Reference
JCM-158	MC4100 *araR/-*		[1]
AK-265	MC4100 *araR/- λ att (PrprA-lacZ)*	WT	[2]
AK-266	AK-265 Δ*rcsF*	Δ*rcsF*	[2]
AK-626	AK-265 *bamE::kan*		This study
AK-790	AK-265 Δ*bamE*	Δ*bamE*	This study
AK-688	AK-265 Δ*rcsF* Δ*bamE*		This study
AK-1214	AK-265 *bamB::kan*	*bamB::kan*	This study
AK-1215	AK-265 Δ*rcsF bamB::kan*		This study
AK-1217	AK-265 Δ*bamE bamB::kan*	Δ*bamE bamB::kan*	This study
AK-1218	AK-265 Δ*bamE bamB::kan* Δ*rcsF*	Δ*bamE bamB::kan* Δ*rcsF*	This study
AK-121	*bamA101*		[3]
AK-271	AK-265 *bamA101*		[4]
MT-100	AK-265 *bamA101 yaeH::Cm*	*bamA101*	This study
MT-102	AK-265 *bamA101 yaeH::Cm* Δ*rcsF*		This study
MT-104	AK-265 *bamA101 yaeH::Cm* Δ*bamE*	Δ*bamE bamA101*	This study
MT-106	AK-265 *bamA101 yaeH::Cm* Δ*bamE*Δ*rcsF*	Δ*bamE bamA101* Δ*rcsF*	This study
DPR-909	JCM-158 *bamD(R197L) nadB*::Tn10		[5]
MT-89	AK-265 *bamD(R197L) nadB*::Tn10	*bamD(R197L)*	This study
AK-1232	AK-265 *bamD(R197L) nadB*::Tn10 Δ*rcsF*		This study
AK-627	AK-265 *bamE::Cm*		[2]
MT-171	AK-265 *bamD(R197L) nadB*::Tn10 *bamE::Cm*	*bamE::Cm bamD(R197L)*	This study
MT-173	AK-265 *bamD(R197L) nadB*::Tn10 *bamE::Cm* Δ*rcsF*	*bamE::Cm bamD(R197L)* Δ*rcsF*	This study
MT-161	AK-265 *bamD(R197L) nadB*::Tn10 (*pZS21*)	*bamD(R197L)*// EV	This study
MT-162	AK-265 *bamD(R197L) nadB*::Tn10 (*pZS21::bamD*)	*bamD(R197L)*// *pbamD*	This study
AK-1248	JCM-158 *λ att (PrprA-lacZ) nadA*::Tn10 *yaeH::Cm*	WT *yaeH::Cm*	This study
AK-1249	JCM-158 *λ att (PrprA-lacZ) nadA*::Tn10 *yaeH::Cm bamA101*	*bamA101 yaeH::Cm*	This study
AK-1250	AK-265 *ΔbamD nadA*::Tn10 *yaeH::Cm pZS21::bamD(L13P)*	*bamD(L13P) yaeH::Cm*	This study

AK-1251	AK-271 *ΔbamD nadA*::Tn10 *yaeH::Cm pZS21::bamD(L13P)*	*bamA101 bamD(L13P) yaeH::Cm*	This study
MT-24	JCM-158 *yafC*::Tn10	WT	This study
MT-21	MT-24 *bamA(F494L)*	*bamA(F494L)*	This study
MT-55	MT-24 Δ*bamE*	Δ*bamE*	This study
MT-52	MT-24 *bamA(F494L)* Δ*bamE*	Δ*bamE bamA(F494L)*	This study
MT-66	MT-24 *bamB::kan*	*bamB::kan*	This study
MT-63	MT-24 *bamA(F494L) bamB::kan*	*bamB::kan bamA(F494L)*	This study
MT-165	MT-24 Δ*bamE bamB::kan*	Δ*bamE bamB::kan*	This study
MT-72	MT-24 *bamA(F494L)* Δ*bamE bamB::kan*	Δ*bamE bamB::kan bamA(F494L)*	This study

1. Malinverni, J.C., et al., *YfiO stabilizes the YaeT complex and is essential for outer membrane protein assembly in Escherichia coli.* Mol Microbiol, 2006. **61**(1): p. 151-64.
2. Konovalova, A., A.M. Mitchell, and T.J. Silhavy, *A lipoprotein/beta-barrel complex monitors lipopolysaccharide integrity transducing information across the outer membrane.* Elife, 2016. **5**.
3. Aoki, S.K., et al., *Contact-dependent growth inhibition requires the essential outer membrane protein BamA (YaeT) as the receptor and the inner membrane transport protein AcrB.* Mol Microbiol, 2008. **70**(2): p. 323-40.
4. Konovalova, A., et al., *Transmembrane domain of surface-exposed outer membrane lipoprotein RcsF is threaded through the lumen of beta-barrel proteins.* Proc Natl Acad Sci U S A, 2014. **111**(41): p. E4350-8.
5. Ricci, D.P., et al., *Activation of the Escherichia coli beta-barrel assembly machine (Bam) is required for essential components to interact properly with substrate.* Proc Natl Acad Sci U S A, 2012. **109**(9): p. 3487-91.
